# Crouzon Syndrome: a case report

**DOI:** 10.1186/1757-1146-3-S1-P15

**Published:** 2010-12-20

**Authors:** David Reid, Stuart Morrison

**Affiliations:** 1 University of East London, London, UK

## 

Crouzon Syndrome is a rare genetic disorder resulting from a mutation of the Fibroblast Growth Factor Receptor 2 Gene. The main presenting feature of this syndrome is craniofacial synostosis but multiple physical dysmorphic features have been reported. There is a dearth of literature detailing the presentation of this syndrome in the foot and lower limb. Therefore, this case report will describe the clinical characteristics of a 22 year old female referred for podiatric assessment. It will also explore the possible treatment options considered for this case.

